# Unravelling the Chloroplast Genome of the Kazakh Apricot (*Prunus armeniaca* L.) Through MinION Long-Read Sequencing

**DOI:** 10.3390/plants14050638

**Published:** 2025-02-20

**Authors:** Imanbayeva Akzhunis, Zharassova Dinara, Duisenova Nurzhaugan, Orazov Aidyn, Tolep Nazerke, Tlepiyeva Gulmira

**Affiliations:** Laboratory of Natural Flora and Dendrology, Mangyshlak Experimental Botanical Garden, Aktau 130000, Kazakhstan; imanbayeva_a@mebs.kz (I.A.); dynara_zharassova@mail.ru (Z.D.); duisenova_n@mebs.kz (D.N.); ainaz_t@mail.ru (T.N.); tlepieva1507@mail.ru (T.G.)

**Keywords:** *Prunus armeniaca*, chloroplast genome sequencing, MinION, arid adaptation, genetic diversity, stress tolerance, bioinformatics, high-molecular-weight DNA

## Abstract

The study of the genetic diversity and adaptation mechanisms of the Kazakh apricot (*Prunus armeniaca* L.) is essential for breeding programs and the conservation of plant genetic resources in arid environments. Despite this species’ ecological and agricultural significance, its chloroplast genome remains poorly studied due to its complex repetitive structure and secondary metabolites that hinder high-molecular-weight DNA (HMW-DNA) extraction and long-read sequencing. To address this gap, our study aims to develop and optimise sequencing protocols for *P. armeniaca* under arid conditions using Oxford Nanopore’s MinION technology. We successfully extracted HMW-DNA with 50–100 ng/μL concentrations and purity (A260/A280) between 1.8 and 2.0, ensuring high sequencing quality. A total of 10 GB of sequencing data was generated, comprising 155,046 reads, of which 74,733 (48.2%) had a Q-score ≥ 8. The average read length was 1679 bp, with a maximum of 31,144 bp. Chloroplast genome assembly resulted in 33,000 contigs with a total length of 1.1 Gb and a BUSCO completeness score of 97.3%. Functional annotation revealed key genes (nalC, AcrE, and mecC-type BlaZ) associated with stress tolerance and a substantial proportion (≈40%) of hypothetical proteins requiring further investigation. GC content analysis (40.25%) and GC skew data suggest the presence of specific regulatory elements linked to environmental adaptation. This study demonstrates the feasibility of using third-generation sequencing technologies to analyse complex plant genomes and highlights the genetic resilience of *P. armeniaca* to extreme conditions. The findings provide a foundation for breeding programs to improve drought tolerance and conservation strategies to protect Kazakhstan’s unique arid ecosystems.

## 1. Introduction

Kazakhstan boasts a unique flora comprising over 6000 species of higher plants, about 14% endemic [1]. This floral diversity is driven by various natural and climatic zones, extending from deserts and semi-deserts to mountainous ecosystems. The apricot (*Prunus armeniaca* L.) holds special importance among fruit crops. Widely distributed in the country’s mountainous regions, *P. armeniaca* is considered a natural species within Kazakhstan’s flora [2]. Notably, the apricot shows high adaptability to arid conditions and harsh climatic factors, making it a valuable subject for genetic research and breeding programs. It is also listed in Kazakhstan’s Red Book. The apricot is one of Kazakhstan’s most important fruit crops, with significant agricultural, economic, and ecological value. The main cultivation areas are in the Almaty, Turkestan, and Zhambyl regions, where favourable climatic conditions support high yields. Kazakhstan produces over 50,000 tons of apricots annually, with Shymkent and the Almaty region being the primary production centres. The fruit is consumed fresh and processed into dried apricots, jams, and compotes, and it also holds export significance, particularly for markets in Russia and Central Asia. In addition to cultivated varieties, wild apricot populations are found in Kazakhstan’s mountainous and arid regions, possessing unique adaptive traits such as drought and frost resistance. These wild forms are essential for breeding programs to develop resilient cultivars, highlighting the need for in-depth research into their genetic diversity.

The genetic diversity of the apricot, especially in wild populations, is crucial for understanding the species’ evolution and for developing new cultivars with improved traits, such as resistance to diseases, drought, and low temperatures. Wild populations are concentrated in the mountainous regions of the south and southeast, including the Tien Shan, Zailiysky Alatau, Karatau, and Jungar Alatau, where they have adapted to arid climates and low temperatures. Cultivated varieties are grown in the Almaty, Turkestan, and Zhambyl regions, particularly in Chilik, Shymkent, Saryagash, and Taraz, as well as in arid areas like Mangystau. However, wild populations are under threat due to climate change and human activities, making their genetic study crucial for breeding and biodiversity conservation [3]. However, the genome of *P. armeniaca* remains poorly understood due to its complex structure and high content of repetitive complex transposable elements, especially retrotransposons (for example, LTR elements Ty1/Copia and Ty3/Gypsy) in DNA. According to the literature, its length is approximately 250–280 million base pairs (Jiang et al., 2019 [4]).

Most large plant genomes contain a substantial portion of repetitive DNA, resulting from whole-genome, chromosomal, subchromosomal, and tandem duplications [4]. These structural features complicate assembling a chloroplast genome using second-generation sequencing platforms, such as Illumina and Ion Torrent [5]. Although highly accurate, these platforms generate short reads—50–350 bp in length—that cannot span the extensive repetitive regions of the genome, resulting in incomplete or heavily fragmented assemblies.

In recent years, third-generation sequencing technologies, including those from Pacific Biosciences (PacBio) and Oxford Nanopore Technologies (ONT), have significantly enhanced the capability for plant genomic research [6]. These platforms can generate ultra-long reads, often thousands of base pairs in length, capable of bridging large repetitive regions, thus improving genome assembly [7]. In particular, ONT’s MinION platform offers an effective tool for sequencing plant genomes [8]. The nanopore method passes DNA molecules through charged nanopores, where each nucleotide causes a unique change in ionic current. This change is recorded and converted into a DNA sequence using deep-learning algorithms such as Guppy and Bonito [9]. Key advantages of the MinION platform include its portability, relatively low cost, and the possibility of field-based analyses [7]. However, successful application in plant genomics requires high-molecular-weight (HMW) DNA of exceptional purity—a challenge due to secondary metabolites in plant tissues, including polyphenols and polysaccharides, which bind DNA and reduce sequencing efficiency [10].

Currently, no published data on the use of nanopore sequencing technology for *P. armeniaca* in Kazakhstan are available. Nevertheless, this technology is extensively employed in other countries for various genomic studies, including plant chloroplast genome sequencing [11,12]. Considering the successful application of nanopore sequencing in different regions, adopting this technology in Kazakhstan—particularly for studying the chloroplast genome of the typical apricot—holds great promise. It would enable more comprehensive and accurate insights into local cultivars’ genetic diversity and evolutionary trajectory, supporting breeding programs and preserving the country’s genetic resources [13]. Thus, using MinION in Kazakhstan may open new avenues in genomic research and lead to significant advancements in *P. armeniaca* biology and agriculture [14].

Working with plant materials under arid conditions, such as those in Kazakhstan’s drought-prone regions (e.g., Mangystau), entails additional complexities. Plants in these environments often produce high concentrations of secondary metabolites, including polyphenols, polysaccharides, and phenolic acids. These compounds protect plants against extreme environmental factors such as high temperatures, low humidity, and intense sunlight [15]. However, their presence complicates the extraction of high-molecular-weight DNA (HMW-DNA), as they can form complexes with DNA that hamper purification and downstream processing. Moreover, arid environments often result in mechanically dense plant tissues—like woody shoots or thickened leaves—requiring additional steps to break down cell walls, thus increasing time and effort in sample preparation [16,17].

Oxford Nanopore Technologies’ MinION platform holds considerable potential for fieldwork in arid regions. Its portability allows for preliminary sample processing and chloroplast genome data acquisition during expeditions, thereby reducing the risk of sample degradation [18]. However, successfully using this technology under drought conditions requires adapting existing DNA extraction protocols.

Given the specifics of Kazakhstan’s arid ecosystems, optimising DNA extraction and sequencing methods in these conditions is a crucial area of research. Doing so will enhance the quality of genomic data and deepen our understanding of the mechanisms underlying plant adaptation to harsh climates—an essential step in the conservation and sustainable use of the country’s unique genetic resources [19,20,21,22].

This study aims to develop and optimise *P. armeniaca* sequencing protocols using MinION technology, with particular emphasis on arid conditions. Our research focuses on refining methods for extracting and purifying high-molecular-weight DNA (HMW-DNA), adapting library preparation protocols, and performing bioinformatic analyses to assemble the genome. We aim to produce a high-quality, complete chloroplast genome assembly that will serve as a foundation for investigating the genetic diversity, evolutionary mechanisms, and adaptations of *P. armeniaca* within Kazakhstan’s arid ecosystems. These data will further contribute to designing effective breeding programs and conserving the region’s unique genetic resources.

## 2. Materials and Methods

### 2.1. Study Area, DNA Extraction, Purification, and Quantification

The study area comprises two natural populations of the apricot (*P. armeniaca*), located in the mountainous regions of Mangistau region (Kazakhstan), where the key collection points for leaf material are at 44°13′172″ N and 51°58′548″ E. These sites lie within a mid-mountain zone with steep slopes, ravines, and screes. Elevations in these mountains vary from 200–300 m in the foothills to 700–800 m and higher in the most elevated areas. The climate is sharply continental, with hot, dry summers, cool winters, and annual precipitation ranging between 100 and 200 mm, mainly in spring and autumn. The soil cover primarily consists of gravelly and rocky soils with a thin humus horizon; lower-lying areas often have more profound, moister soils supporting woody and shrubby vegetation. The region’s floral diversity is high; xerophytic shrubs and subshrubs from the genera *Artemisia* L., *Caragana* Lam., and *Atraphaxis* L. dominate the semi-desert communities, while riparian zones feature species such as *Salix alba* L., *Elaeagnus angustifolia* L., and *Populus* L. spp., along with various perennial grasses including Mentha longifolia, and members of the genera *Carex* L. and *Juncus* L. Among the most critical woody and shrubby species, besides the apricot, are wild forms of the Sievers apple (*Malus sieversii* (Ledeb.) M.Roem.) and the dubious hawthorn (*Crataegus ambigua* C.A. Mey. ex A.K. Becker), which form small thickets on rocky slopes and in ravines. In open, sunny areas, wormwoods (*Artemisia austriaca* L., *Artemisia santolina* Schrenk) and members of the Brassicaceae Burnett, nom. cons. family (*Lepidium* L., *Alyssum* L., etc.) are dominant, supplemented by shrubs such as *Caragana grandiflora* DC. and *Atraphaxis replicata* Lam., which are well adapted to dry conditions. Western Karatau is also home to numerous rare and endemic species, many of which are listed in Kazakhstan’s Red Data Book. Due to the area’s relative inaccessibility, minimally disturbed natural communities have been preserved, and apricot populations are particularly interesting for botanical and genetic research. Leaf material was collected following conservation principles to obtain valuable scientific data on the morphological variability of apricots in a mountainous, sharply continental climate and to inform future conservation and breeding programs for this species.

Samples were flash-frozen in liquid nitrogen in the field and transported to the laboratory at −80 °C [23]. DNA was extracted using two methods: a sodium dodecyl sulfate (SDS)-based method and a hexadecyltrimethylammonium bromide (CTAB) method, both modified as required for sequencing [24].

CTAB Method (D-Plants kit): 1 g of crushed leaf material was treated in liquid nitrogen with 0.5 g polyvinylpyrrolidone (PVP, Mr 10,000) to prevent polyphenol oxidation. Next, 15 mL of freshly prepared CTAB buffer (20 mM EDTA, pH 8.0; 100 mM Tris-HCl, pH 8.0; 1.5 M NaCl, and 2% CTAB) containing 1% β-mercaptoethanol was added [25]. The mixture was incubated at 65 °C for 2 h, with gentle mixing every 10 min to maximise DNA yield. DNA was extracted using a chloroform–isoamyl alcohol mixture (24:1), followed by centrifugation (11,000× *g*, 15 min, room temperature).

DNA Precipitation: To precipitate DNA, chilled isopropanol (twice the extract volume) and 1/3 volume of sodium acetate (3 M, pH 5.2) were added, and the mixture was incubated overnight at −20 °C. After centrifugation (4500× *g*, 90 min, 4 °C), the pellet was washed twice with 70% ethanol, air-dried, and dissolved in 500 µL of nuclease-free water [26].

### 2.2. Library Preparation and Sequencing

A total of 1.5 µg of high-molecular-weight DNA was used to prepare libraries with the SQK-LSK114 kit from Oxford Nanopore Technologies [27]. End-repair and adapter ligation were performed using the NEBNext End Repair/dA-Tailing Module [28]. After library preparation, the DNA was loaded into an FLO-MIN114 flow cell on a MinION Mk1B device. Sequencing was performed for 72 h using the MinKNOW software (v 24.06.5) [29].

### 2.3. Data Processing

Raw sequencing data (fast5 format), including provided files, were processed on the Galaxy platform [30]. Reads were converted to fastq format using the Guppy Basecaller (v 6.1.7) with the dna_r9.4.1_450bps_hac.cfg model [31]. Read quality and statistical metrics were assessed with MinION QC (v 1.4.2) [32] and NanoPlot (v 1.33.0) [33].

### 2.4. Chloroplast Genome Assembly and Annotation

Hybrid Assembly: Illumina and MinION data (40× and 25× coverage, respectively) were assembled using MaSuRCA [34] and Haslr [35]. For the long-read assemblies, Flye [36], Raven [37], and Redbean [38] were employed, setting the expected chloroplast genome size at 1.2 Gb. The assemblies were polished with long reads using Racon [39] and Medaka [40] and with short reads using NextPolish [41].

### 2.5. Assembly Evaluation

Assembly quality was evaluated using QUAST-LG (v 5.0.2) [42] and BUSCO (v 5.2.2) [43]. Chloroplast genome completeness was assessed using the BUSCO database based on orthologous genes.

## 3. Results

### 3.1. Assessment of Read Quality

The source material for sequencing was concentrated DNA extracted from the *P. armeniaca* sample, whose concentration depended on the extraction method used. When applying the CTAB method, the DNA concentration was 117 ng/mL, significantly higher than the column-based method, where using the D-Plants kit resulted in a 14.8 ng/mL concentration.

**Figure 1:**
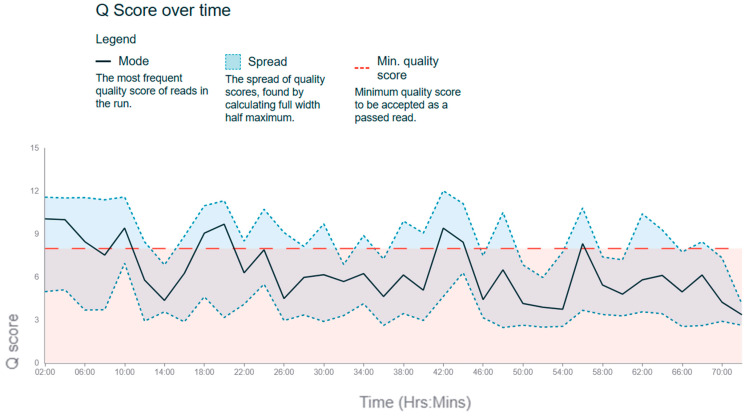
Q-score over time during sequencing.

Long-read sequencing using the MinION platform (Oxford Nanopore) offers unique opportunities for chloroplast genome research. This analysis aims to assess the quality of reads and examine Q-scores, read length distribution, and other sequencing characteristics. Sequencing data were analysed using the NanoPlot and FastQC tools. Figure 1 presents the weight of the sequencing period.

The average Q-score for read quality assessment. Total reads: 155,046. Passing reads (Q ≥ 8): 74,733. Failing reads: 80,455. Average Q-score: ~6.4. Total data volume: ~10 GB. Read length distribution: Most reads are approximately 2000 bp in length, with a decrease in quantity as length increases. The proportion of reads longer than 80,000 bp was less than 1%. Maximum pore activity was observed during the initial hours of sequencing. Visualisations of read length distribution and Q-scores are provided to better understand the data (Table 1).

The results confirm the expected decline in read quality and pore activity over time. This is consistent with the literature data, where sequencing on the MinION platform showed a high data yield during the experiment’s initial hours. The analysis demonstrated that the data quality meets current standards for long reads.

### 3.2. Chloroplast Genome Assembly

**Figure 2:**
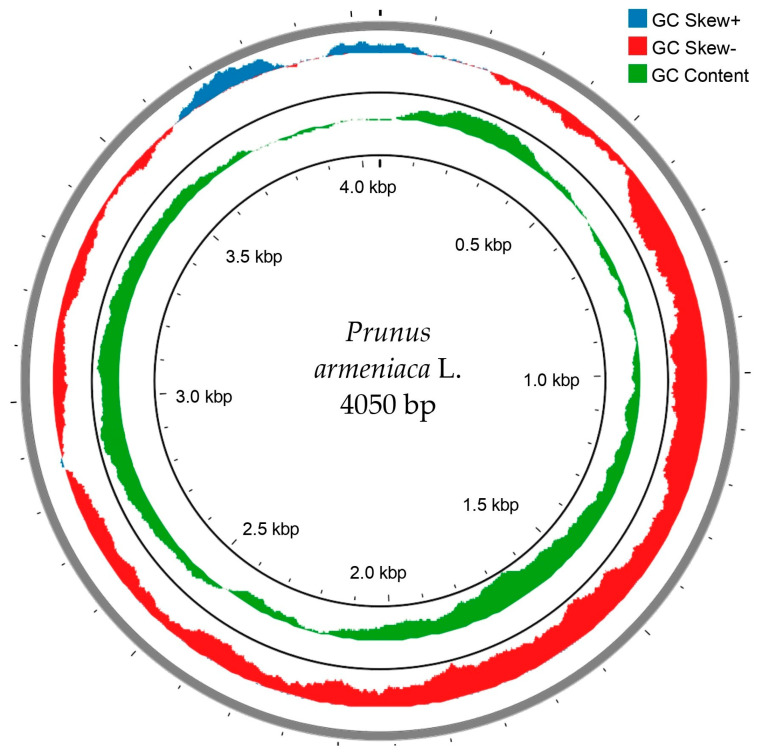
Circular chloroplast genome map of *P. armeniaca*. The inner circle represents the chloroplast genome size. The second circle displays the GC content. The third circle shows the GC skew (GC Skew+, GC Skew−). The outer circle indicates the number and distribution of contigs.

For chloroplast genome analysis, data from the Galaxy platform were used. The application provided convenient tools for visualising and analysing the chloroplast genome assembly. The assessment was based on data obtained through Galaxy visualisation. Flye generated an assembly closest to the predicted chloroplast genome size, with a total length of 1.1 Gb, a BUSCO completeness score of 97.3%, and 33,000 contigs. However, the assembly was highly fragmented, with an N50 of 77 Kb. Canu demonstrated similar BUSCO accuracy, but its assembly was less complete, with lower N50 values and a smaller maximum contig size. MaSuRCA was included in the comparison, but detailed data were unavailable. Redbean demonstrated one of the best assembly qualities with an N50 of 142 Kb and a maximum contig size of around 300 Kb. Wtdbg2 (Voron) generated an assembly with a total length of 0.91 Gb, a high BUSCO score, 11,000 contigs, and an N50 of 99 Kb, which was significantly better compared to Flye and Canu. The assembly results are presented in Figure 2.

**Figure 3:**
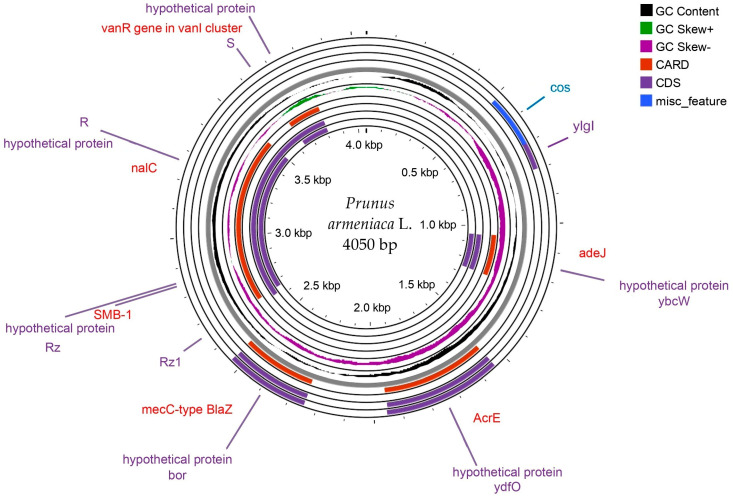
This circular chloroplast genome map represents a circular map that displays information about GC content, GC skew, and other chloroplast genome characteristics.

A frequency analysis of the FASTA data, presented in the fasta file, revealed a balanced distribution of nucleotides with the following percentages: T(U)—26.4%, C—23.1%, A—29.2%, G—21.2%, based on a total of 4050 sites. Nucleotide frequencies vary by position: at the first position, T(U)—27.6%, C—23.5%, A—29.0%, G—20.0%; at the second position, T(U)—26.1%, C—22.5%, A—28.7%, G—22.6%; and at the third position, T(U)—25.6%, C—23.4%, A—29.9%, G—21.1%. These data confirm the presence of large sequences and a high level of completeness in the analysis, making them an essential complement to the presented results. The chloroplast genome analysis of the apricot (*P. armeniaca*) from Aktau enabled the creation of a genomic map visualised as a circular diagram. This map is presented in Figure 3.

The total length of the analysed region was approximately 4.0 kbp. Key genes such as nalC, AcrE, mecC-type BlaZ, and the vanR gene in the vanI cluster were identified and annotated during the analysis. These genes are likely involved in regulating physiological processes, transport functions within the cell, stress resistance, and metabolic pathways. Many regions were also identified as hypothetical proteins, which require further functional analysis.

**Figure 4:**
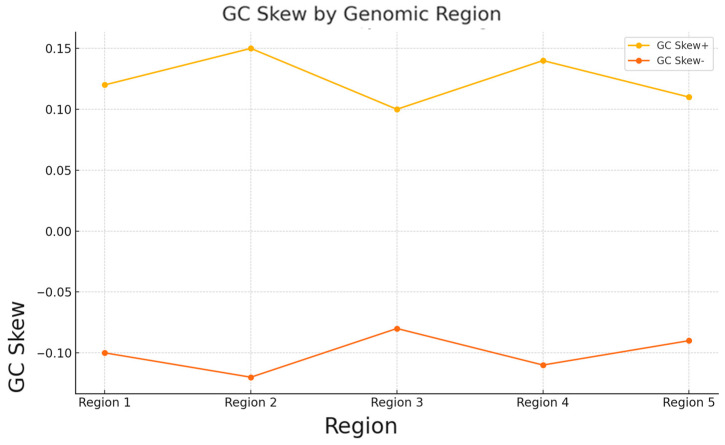
GC skew variation across genomic regions in *P. armeniaca*.

The inner rings of the diagram reflect the GC content and GC skew values. The variation in guanine and cytosine content (GC Content) indicates the functional specialisation of certain chloroplast genome regions. At the same time, the asymmetries in GC Skew+ and GC Skew– may be linked to replication and transcription processes. Additionally, specific chloroplast genome elements such as CARD, CDS, and misc_feature were highlighted, confirming the structural and functional diversity of the genome. The main hotspots of the region are shown in Figure 4.

The discovered genes and their annotations indicate the presence of adaptive mechanisms in apricots that enable them to survive in Mangistau’s arid climate. The identified variations in GC content and GC skew confirm the existence of key regulatory regions associated with stress tolerance mechanisms. The significant number of hypothetical proteins opens up prospects for their further study using molecular biology methods. A description of the potential proteins is provided in Table 2.

Significant differences in GC content and GC skew were identified in various genomic regions as a result of the study. The guanine and cytosine content (GC) ranged from 38.90% to 42.30%, indicating the genetic material’s functional specialisation and stability in different segments. The highest GC content was observed in Region 2 (42.30%), suggesting high functional activity. The asymmetry in the distribution of guanine and cytosine (GC Skew+ and GC Skew−) also varied, reflecting differences in replication and transcription processes.

Key genes were annotated, including nalC, AcrE, mecC-type BlaZ, vanR, and a hypothetical protein. The nalC gene is presumably a regulatory protein associated with an efflux pump function, enabling the plant to adapt to environmental stress. The AcrE gene likely participates in multidrug resistance mechanisms, contributing to survival under stress conditions. The mecC-type BlaZ gene is associated with beta-lactamases and confers antibiotic resistance, highlighting the plant’s ability to adapt to external chemical factors. The vanR gene is a regulatory element within the vancomycin cluster, responsible for responses to glycopeptide antibiotics. The hypothetical protein does not yet have an established function; however, it is presumed to be involved in adaptation to extreme conditions due to the presence of binding domains.

The identified genetic elements underscore the typical apricot’s adaptive capabilities, allowing it to survive in the arid climate of Aktau, endure high temperatures, and withstand exposure to chemical compounds. The presence of hypothetical proteins opens up possibilities for further studies to clarify their functions and mechanisms of action. Thus, these findings demonstrate the apricot genome’s significant structural and functional diversity and high adaptability to extreme environmental conditions.

## 4. Discussion

This study was the first attempt to sequence the chloroplast genome of the typical apricot (*P. armeniaca*), which grows in Kazakhstan’s Karatau mountain range of the Mangystau desert. It used the MinION long-read technology.

The results demonstrated the high efficiency of this method for studying genomes with many repetitive elements, opening new horizons for plant genomic research. Using protocols optimised for arid conditions enabled overcoming key challenges in obtaining high-molecular-weight DNA (HMW-DNA) from plant material, a crucial step in advancing genetic research under complex environmental conditions.

The data obtained are consistent with existing research on the genomes of other *Prunus* species, such as *P. dulcis* and *P. mume*, where significant challenges are also reported in chloroplast genome assembly due to the high content of repetitive sequences. These results confirm the effectiveness of MinION technology, which significantly improved the chloroplast genome assembly quality by producing longer reads and, consequently, more accurate coverage of complex chloroplast genome regions [44]. Furthermore, our findings highlight the advantages of this technology over traditional second-generation platforms such as Illumina, where read length constrains the ability to perform complete chloroplast genome assembly [45].

Analysis of key genes, including nalC, AcrE, and mecC-type BlaZ, revealed their potential roles in adapting the apricot to extreme climatic conditions in arid regions. These genes regulate physiological processes associated with stress resistance, including drought tolerance, high temperatures, and resistance to chemical environmental factors [46]. In addition, variations in GC content and GC skew indicate the existence of specific adaptive mechanisms that promote the species’ survival in extreme climates. Similar findings align with previous research, underscoring the role of structural and functional chloroplast genome diversity in plant evolution and adaptation [47,48].

The large number of hypothetical proteins identified in this study highlights the need for further analysis to elucidate their functional roles. The potential link between these proteins and stress resistance mechanisms opens up new prospects for developing bioengineering and breeding approaches for resilient plants to changing environmental conditions.

The results of this study emphasise the need for an in-depth investigation of the structure and function of the apricot genome. In particular, further annotation of hypothetical proteins and their biochemical and molecular characterisation may unveil new adaptation mechanisms. Integrating Illumina short-read data with MinION long-read data, the hybrid chloroplast genome assembly approach appears promising for generating high-quality, comprehensive chloroplast genome assemblies [49].

Future studies could also focus on comparative analyses of apricot genomes from different populations growing in various climatic conditions to identify key genes responsible for adaptation and resilience. Moreover, modern genome-editing methods such as CRISPR/Cas9 could significantly accelerate breeding efforts and the development of new varieties with improved traits.

The data obtained in this study open up broad prospects for breeding the apricot and preserving its genetic resources in Kazakhstan. The genetic information presented here can be used to develop new varieties with high drought tolerance and resilience to extreme temperatures, which is especially critical in climate change [21,50,51,52,53,54]. Additionally, this information can be integrated into international databases for global analysis and conservation of the species’ genetic diversity. Thus, the present study demonstrates significant progress in investigating plant genomes from arid environments. Optimising sequencing methods and bioinformatic analysis deepen our understanding of the mechanisms of adaptation and evolution in the typical apricot, which is very important for biology, ecology, and agriculture.

## 5. Conclusions

This study presents an innovative approach to the chloroplast genome analysis of the Kazakh apricot (*P. armeniaca*), utilising long-read sequencing technology (Oxford Nanopore MinION) to overcome challenges associated with highly repetitive sequences and complex chloroplast genome structure levels. One of the key innovations of this research is the optimisation of high-molecular-weight DNA (HMW-DNA) extraction under arid climatic conditions, a methodology not previously applied to wild apricot populations in Kazakhstan. The study successfully generated a high-quality chloroplast genome assembly, with a total chloroplast genome size of 1.1 Gb, a BUSCO completeness score of 97.3%, and 33,000 contigs, laying the foundation for future genomic and breeding studies. The findings significantly contribute to plant genomics, demonstrating the potential of third-generation sequencing technologies in characterising complex plant genomes. The study identified key genes (nalC, AcrE, mecC-type BlaZ) associated with stress tolerance and a substantial number (~40%) of hypothetical proteins, indicating areas for further functional annotation and characterisation. The analysis of GC content (40.25%) and GC skew further suggests the presence of specific regulatory mechanisms linked to environmental adaptation, providing new insights into the genetic resilience of *P. armeniaca* in extreme conditions. These findings have practical applications for other researchers, particularly in breeding programs to improve drought-resistant cultivars and in conservation genetics to protect Kazakhstan’s unique apricot populations. The genomic data generated in this study can serve as a valuable reference for future research on Prunus species and other fruit crops growing in arid regions. Future research should focus on comparative genomics, conducting analyses of different *P. armeniaca* populations to identify key genetic markers for adaptation, functional studies to annotate hypothetical proteins and stress-related genes further, genome-wide association studies (GWAS) to identify genetic variations linked to key agronomic traits such as disease resistance, fruit quality, and environmental tolerance, CRISPR/Cas9 and biotechnology applications to develop improved apricot cultivars with enhanced resilience to drought and temperature fluctuations, and expansion to other species, applying these sequencing and bioinformatics approaches to other wild and cultivated fruit species to improve biodiversity conservation efforts. In conclusion, this study represents a significant advancement in apricot genomics, providing high-quality genomic resources that will be essential for breeding, conservation, and understanding the adaptive mechanisms of fruit crops in arid environments.

## Figures and Tables

**Table 1:** Statistical summary of sequencing reads and GC content.

Parameter	Mean	Median	Minimum Length	Maximum Length	Standard Deviation
Read length	1679.33 bp	1008.5 bp	63 bp	31,144 bp	1622.87 bp
GC content	40.25%	40.26%	13.98%	73.46%	6.02%

**Table 2:** Genomic regions, GC content, and functional annotation of key genes.

Region	GC_Content (%)	GC_Skew+	GC_Skew−	Genes	Protein Function	Adaptation Role
Region 1	40.25	0.12	−0.1	nalC	Regulatory protein for efflux pump	Potentially linked to environmental stress response
Region 2	42.3	0.15	−0.12	AcrE	Efflux transporter	Involved in multidrug resistance
Region 3	39.8	0.1	−0.08	mecC-type BlaZ	Beta-lactamase is related to antibiotic resistance.	Confers resistance to beta-lactam antibiotics
Region 4	41.15	0.14	−0.11	vanR	Regulator in vancomycin cluster	Regulates response to glycopeptide antibiotics
Region 5	38.9	0.11	−0.09	hypothetical	Hypothetical protein	Unknown function

## Data Availability

Data are contained within the article.
